# Efficacy and safety of multi-loop traction device-assisted colorectal endoscopic submucosal dissection: Multicenter randomized clinical trial

**DOI:** 10.1055/a-2466-0718

**Published:** 2025-04-04

**Authors:** Mamoru Ito, Yuko Miura, Yasuhiko Mizuguchi, Hiroto Furuhashi, Yosuke Tsuji, Hiroyuki Takamaru, Naoto Tamai, Mitsuhiro Fujishiro, Yutaka Saito, Kazuki Sumiyama

**Affiliations:** 1 12839 Department of Endoscopy, The Jikei University School of Medicine, Minato-ku, Japan; 2 13143 Department of Gastroenterology, The University of Tokyo, Bunkyo-ku, Japan; 3 68380 Endoscopy Division, National Cancer Center Hospital, Chuo-ku, Japan

**Keywords:** Endoscopy Lower GI Tract, Polyps / adenomas / ..., Colorectal cancer, Endoscopic resection (polypectomy, ESD, EMRc, ...)

## Abstract

**Background and study aims:**

The multi-loop traction device (MLTD) facilitates optimal visualization of the submucosa throughout endoscopic submucosal dissection (ESD). This trial aimed to assess the efficacy of MLTD for colorectal ESD.

**Patients and methods:**

We conducted a multicenter, open-label, randomized controlled trial involving patients with colorectal lesions ≥ 20 mm suspicious for noninvasive carcinoma. Participants were randomly assigned in a 1:1 ratio to undergo ESD with MLTD (MLTD-ESD group) or ESD without any traction device (control group). Endoscopists were allowed to convert treatments if dissection became challenging for 10 minutes. The primary endpoint was dissection speed; secondary endpoints included technical success rate and adverse events (AEs).

**Results:**

A total of 108 participants were randomized to the MLTD-ESD group (n = 53) and the control group (n = 55). There was no statistically significant difference in median dissection speed between the MLTD-ESD group and the control group (14.8 mm ^2^ /min; interquartile range [IQR] 8.9–23.9 mm ^2^ /min vs. 13.3 mm ^2^ /min; IQR 8.9–18.8 mm ^2^ /min) ( *P* = 0.33). The technical success rate was significantly higher in the MLTD-ESD group (96.2%) compared with the control group (71.0%) ( *P* < 0.0001). All technical failures were due to treatment conversions. No significant difference was observed in AEs. Subgroup analysis revealed that experts in the MLTD-ESD group had faster dissection speed than controls (21.6 mm<sup>2</sup>/min; IQR 15.5–28.8 mm ^2^ /min vs. 14.4 mm ^2^ /min; IQR 9.9–21.2 mm ^2^ /min) ( *P* = 0.009).

**Conclusions:**

This multicenter randomized trial demonstrated that use of MLTD did not significantly increase dissection speed for colorectal ESD. Treatment conversions may have influenced the primary endpoint, and further investigation is warranted.

## Introduction

Endoscopic submucosal dissection (ESD) is a minimally invasive endoscopic resection technique allowing noninvasive superficial gastrointestinal neoplasia to be removed en bloc regardless of tumor size 1 . The most labor-intensive step is dissecting thin and soft submucosal connective tissues using diathermy with meticulous endoscopic maneuvers, which may take longer procedure time and carry higher risks of adverse events (AEs) such as severe bleeding and perforation 2 . A major cause of technical challenges associated with submucosal dissection is inadequate visualization of submucosal tissue planes 3 . A variety of traction techniques, using gravitational traction 4 , hood attachment 5 and traction devices, have been widely used to facilitate ESD by deflecting the mucosal overlay away from the dissection plane 6 7 8 9 10 11 . A systematic review that covered 3,134 patients who underwent gastrointestinal ESD 12 proved that anchor-guided ESD, the double endoscope method, and clip-assisted methods accomplished shortener procedure times, higher rates of R0 resection, and reduced occurrence of bleeding and perforation. Prior multicenter randomized controlled trials (RCTs) have confirmed that use of traction devices hastened the submucosal dissection process in the esophagus and stomach 13 14 , although the advantage in the stomach was observed only for lesions in the greater curvature of the upper and middle portions of the corpus.

Because of the redundant anatomy and thinner wall of the colon, colon ESD is considered technically more challenging than upper gastrointestinal ESD. Therefore, it has been strongly recommended that trainees perform enough gastric ESDs before undertaking colorectal ESD in Japanese institutions 15 16 17 . However, the same training program may not be applicable to Western endoscopists, given the low prevalence of upper gastrointestinal cancers. Development of an optimal traction technique for the colon would be required to globally propagate the concept of en bloc resection and ESD. A series of single-center RCTs have demonstrated that use of traction devices facilitated colorectal ESD, reducing procedure time 6 8 11 . Meanwhile, the latest multicenter RCT (CONNECT-C study) 18 comparing traction assisted ESD using different types of traction devices and traditional non-assisted ESD showed no statistical difference in procedure time.

We developed a novel soft resin traction device comprising three interconnected loops made from ultrathin linear low-density polyethylene (multi-loop traction device [MLTD]; Boston Scientific Co. Ltd., Tokyo, Japan) 19 . Device delivery, deployment, and fixation of the traction device can be performed with regular endoclips via an endoscope accessory channel without need for scope retrieval. We surmised that the through-the-scope traction device would be more desirable for colon ESD. A pilot study was conducted comparing MLTD-assisted colorectal ESD in novices with conventional colorectal ESD in experts 20 . The results revealed no statistically significant difference in procedure time, dissection speed, or AEs, suggesting its usefulness for safe introduction of colorectal ESD. We hypothesized that MLTD would facilitate efficient traction and enhance dissection speed in colorectal ESD. In this study, the technical advantage of procedure time reduction with MLTD vs colorectal ESD was evaluated in a multicenter RCT.

## Patients and methods

### Trial design

A multicenter, open-label, parallel group RCT was conducted at four high-volume academic centers in Japan. The study protocol was registered and disclosed online before recruitment of the first participant. There were no significant changes in the methods, including eligibility criteria, interventions, and outcomes, after trial commencement. We obtained written informed consent from all participants. This study followed ethical guidelines for medical and health research involving human subjects set by the Ministry of Health, Labor and Welfare, Japan, the 1964 Declaration of Helsinki, and its later amendments. Approval was obtained from the institutional review board of each participating facility (study protocol No. 33–281).

### Participants

The participants were adults aged 20 years or older undergoing colorectal ESD for lesions ≥ 20 mm suspicious for noninvasive carcinoma. Patients with the following criteria were excluded: refusal to provide consent, conditions precluding perioperative antithrombotic therapy management, severe organ failure, blood coagulation disorders, pregnancy, urgent and life-threatening condition, and ineligibility to participate as judged by the operator. Lesions involving the terminal ileum, appendiceal orifice, or diverticulum, and recurrent lesions on the scar after endoscopic treatment were excluded. In participants with more than two eligible lesions, the most proximal lesion was selected for the trial.

### Randomization

Study participants were recruited at four tertiary medical centers in Japan (The Jikei University Hospital, Tokyo, National Cancer Center Hospital, Tokyo, The University of Tokyo Hospital, Tokyo, and The Jikei University Kashiwa Hospital, Chiba). Patient registration and randomization was performed by the INDIC-Cloud automated computer-generated randomization system (INDICE-Cloud), managed by UMIN Internet Data and Information system for Clinical and Epidemiological research. Patients were randomly assigned to either the MLTD-ESD group or the control group in a 1:1 ratio. Allocation was determined using the minimization method with stratification based on the institution. Participants and endoscopists were unblinded in the study, whereas treatment data and case report forms were collected and concealed throughout the study, except from the independent data manager approved by the institutional review board.

### Multi-loop traction device

**Fig. 1:**
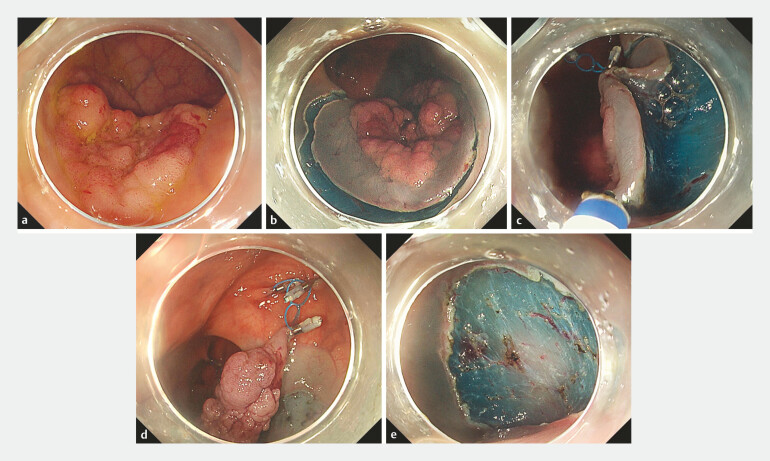
Procedure steps in the MLTD-ESD group. **a** Colorectal lesion suspicious for early colon carcinoma. **b** Circumferential mucosal incision. **c** Traction clip anchored to the mucosal edge of the circumferentially isolated mucosa. **d** Additional clipping to readjust traction force. **e** Complete removal of the lesion from the intestinal wall.

The MLTD is composed of three connected loops made of straight-chain low-density polyethylene, with a loop thickness of 0.3 mm and a diameter of 6 mm. It is commercially available in Japan for tissue traction and manipulation during endoscopic procedures. The MLTD is grasped with an endoscopic clip and inserted into the forceps channel for delivery to the gastrointestinal lumen. Traction is provided by clipping one end of the MLTD onto the proximal edge of a circumferentially isolated mucosa and the other end onto the opposite side of the healthy gastrointestinal wall. Direction and traction strength can be modulated by adding a clip to fix the free middle loop of the device onto other sites. The device can be safely and quickly retrieved using a forceps with a specimen at the end of the procedure. The steps in MLTD are presented in Fig. 1 .

### Endoscopists

Endoscopists were required to have performed a minimum of 10 clinical colorectal ESDs to participate in the study. If the primary operator had performed fewer than five MLTDs, an additional endoscopist who had performed at least five MLTDs had to be present.

### Endoscopic procedure

Colorectal ESD was performed under intravenous anesthesia using benzodiazepine sedatives and pethidine hydrochloride. The submucosa was elevated with local injection of 0.2% or 0.4% hyaluronic acid solution. An electrocautery knife (Dual Knife, Olympus Corporation, Tokyo, Japan; Flush Knife BT, FUJIFILM Corporation, Tokyo, Japan; Endosaber, Sumitomo Bakelite Co., Ltd., Tokyo, Japan; or IT-nano, Olympus Corporation, Tokyo, Japan) in conjunction with a high-frequency thermal coagulation device (VIO 3 or VIO 300D; Erbe Elektromedizin GmbH., T Tübingen, Germany) was used for ESD. In the MLTD-ESD group, the MLTD was applied when countertraction was deemed appropriate for dissection. Timing of device attachment (e.g., before circumferential incision, after proximal dissection, after circumferential incision) was left to operator discretion. All MLTDs were affixed to the gastrointestinal wall with either the EZ Clip (Olympus Corporation, Tokyo, Japan) or the SureClip (MicroTech Medical Co., Ltd., Hangzhou, China). In case of unintended detachment of the MLTD, the operator was permitted to reuse it or use another MLTD in case of unintended device detachment. After complete excision of the lesion, the MLTD was severed from the wall with biopsy, grasping, or hemostatic forceps and removed along with the specimen. Postoperative management adhered to the standard treatment protocol at each institution.

### Treatment conversion criteria

Under specific circumstances, operators were given the option to convert treatment to the other group during ESD. In the control group, they were allowed to use the MLTD if device-assisted traction method was deemed appropriate to proceed with the procedure for more than 10 minutes. In the MLTD-ESD group, they were permitted to transition into conventional ESD if removal of the MLTD was considered necessary to carry out the procedure for more than 10 minutes, or if the endoscopist encountered difficulty in attaching the MLTD. Cases in which the MLTD unintentionally detached and the operator chose not to use MLTD to continue the dissection were considered treatment conversion.

### Outcome measures

The primary endpoint was dissection speed (mm ^2^ /min), determined by dividing the area of the resected specimen (mm ^2^ ) by procedure time (min). The area of the resected specimen was calculated using the formula: (major axis of the excised specimen [mm])×(minor axis of the excised specimen [mm])×(3.14/4). Secondary endpoints included procedure time (min), technical success rate, en bloc resection rate, R0 resection rate, intraoperative perforation rate, delayed perforation rate, and delayed bleeding rate. Procedure time was defined as duration from initial submucosal injection to complete dissection of the lesion. Time required for MLTD removal, prophylactic hemostasis, or closure of mucosal defect was not included. Technical success rate was defined as the proportion of cases in which the protocol treatment was completed in each group. Cases in which protocol treatment could not be completed included: conversions from the MLTD-ESD to ESD without traction; cases in the control group in which the MLTD was applied; dissection difficulties, untreatable intraoperative perforation, or a sudden change in patient condition requiring treatment termination; and conversion to endoscopic mucosal resection (EMR). En bloc resection was defined as complete removal of the tumor without piecemeal resection during the endoscopic procedure. Failure of en bloc resection included cauterization for suspected residual tissue at the post-ESD ulcer edge or presence of detected tumor in the biopsy from the residual area. R0 resection was defined as en bloc resection with horizontal and vertical margins free of neoplasia according to pathological diagnosis. Intraoperative perforations were defined as complete penetration of the serosa. Delayed perforations were defined as post-procedure free air confirmed on x-ray or computed tomography (CT) scan. Delayed bleeding was defined as rectal bleeding within 14 days of the procedure with at least one of the following criteria: decrease in hemoglobin levels ≥ 2 g/dL, need for endoscopic hemostasis, transcatheter arterial embolization, surgical hemostasis, or blood transfusion.

The following clinical data were collected post-procedure: age, sex, overall ESD experience of the operator (number of cases: 10–99/100–199/200–499/500–999/1000 or more), handover to a more experienced endoscopist, tumor location, tumor morphology according to Paris Classification (0-Is/IIa/IIb/IIc); laterally spreading tumor classification (LST-G-H/LST-G-Mix/LST-NG-FE/LST-NG-PD); tumor diameter (mm); specimen diameter (mm); treatment conversion; procedure time after conversion (min); muscularis propria injury, defined as partial or complete rupture of inner circular muscle observed during ESD treatment; and final pathological diagnosis according to World Health Organization classification. In the MLTD-ESD group, the following data also were collected: number of MLTDs used; MLTD placement success rate, defined as proportion of MLTDs placed without detachment, relative to total number of MLTDs used; and lesion tissue damage at the MLTD site, as evaluated by endoscopic images or by visual inspection.

### Sample size calculation

A single-center RCT 6 reported an increase in dissection speed of 11.0 mm ^2^ /min increase in dissection speed using S-O clip for colorectal ESD. The S-O clip operates similarly to the MLTD by anchoring one end near the lesion and securing the other to the contralateral wall. Assuming treatment conversions would reduce the difference of dissection speed between the two groups, we hypothesized a 9.9 mm ^2^ /min increase in dissection speed. The standard deviation (SD) was projected to be 17.3 mm ^2^ /min, based on 319 colorectal ESDs performed at The Jikei University Hospital from January 2017 to December 2019. The required sample size was calculated to be 49 patients per group, setting a significance level of 0.05 and a statistical power of 0.80. Considering a 10% dropout rate, 109 patients were needed for recruitment.

### Statistical analysis

Statistical analysis was conducted using a modified intention-to-treat approach. The full analysis set (FAS) included all patients other than those who withdrew from the trial prior to receiving any treatment intervention. Secondary analysis was performed on the per-protocol set (PPS), which included patients who completed the protocol treatment. Exclusions from PPS included MLTD-ESD cases converted to ESD without traction, control cases converted to ESD with MLTD, and conversions to EMR. Missing data were not imputed, and outliers were kept in the analysis.

For continuous variables, descriptive statistics (maximum, median, minimum, quartiles, mean and SD) were computed. For categorical data, frequencies were calculated. Between-group differences in demographic and clinical data were evaluated using Pearson’s chi-square test or Fisher’s exact test for categorical variables and Student’s *t* -test or Wilcoxon rank-sum test for continuous variables. Bonferroni correction was applied for comparisons across multiple categories (i.e., tumor morphology, histological pathology) in the patient background data. For the primary outcome, multiplicity was not an issue, given that there was only one primary outcome measure. Point estimates and 95% confidence intervals (CIs) were calculated for both groups. Differences in dissection speed, total procedure time were evaluated using analysis of covariance (ANCOVA) with the stratification factor (institution). Technical success rate, en bloc resection rate, R0 resection rate, and AEs were evaluated using Pearson's chi-square test or Fisher’s exact test. Two-tailed tests were used for comparing two groups, and *P* < 0.05 was considered statistically significant. There was no interim analysis.

All analyses were performed using R version 4.4.1 (R Foundation for Statistical Computing, Vienna, Austria). Artwork creation was performed using Prism version 9.5.1 (GraphPad Software, LLC, California, United States).

## Results

### Study population

**Fig. 2:**
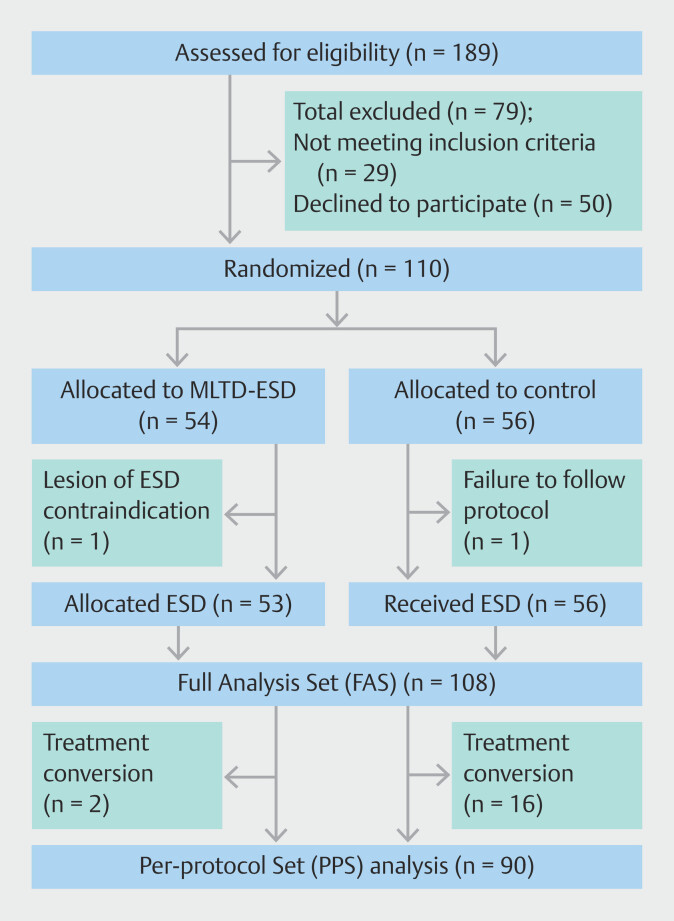
Flowchart of patients through enrollment, randomization, intervention, and analysis.

Patient flow in the trial is shown in Fig. 2 . From May to December 2022, 189 patients were assessed for eligibility. Excluding 50 patients who did not consent and 29 who did not meet the criteria, 110 patients enrolled: 54 in the MLTD-ESD group and 56 patients in the control group. One patient was excluded due to unresectable tumor, and one case failed to adhere to the protocol. The FAS included 53 patients in the MLTD-ESD group and 55 in the control group. Baseline characteristics were similar between the two groups. Median tumor diameters were 34 mm (interquartile range; [IQR] 29–45) in the MLTD-ESD group, and 36 mm (IQR 30–40) in the control group. In the MLTD-ESD group, two patients (4%) required treatment conversions, compared with 16 patients (29%) in the control group ( *P* = 0.005) ( Table 1 ).

**Table:** **Table 1** Clinical data for the full analysis set.

	**MLTD-ESD (n = 53)**	**Control (n = 55)**	***P* value **
Age, years; mean ± SD	63.9 ± 12.9	66.0 ± 13.2	0.37*
Sex, n; male/female	34/19	35/20	0.96*
ESD experience of the operator, n (%)			0.49†
10–99	12	12	
100–199	16	9	
200–499	7	11	
500–999	8	10	
Over 1000	10	13	
Handover to more experienced endoscopist	17% (9/53)	24% (13/55)	0.39†
Tumor location, n (%)			0.27‡
Cecum	6 (11)	11 (20)	
Ascending colon	15 (28)	11 (20)	
Transversus colon	13 (25)	11 (20)	
Descending colon	6 (11)	3 (5)	
Sigmoid colon	4 (7)	11 (20)	
Rectum	9 (17)	8 (14)	
Morphology, n (%)			0.18‡
0-IIa LST-G Homo	6 (11)	10 (18)	
0-IIa LST-G Mix	17 (32)	7 (13)	
0-IIa LST-NG FE	17 (32)	20 (36)	
0-IIa LST-NG PD	7 (13)	11 (20)	
0-IIb	0 (0)	1 (2)	
0-Is	6 (11)	6 (11)	
Tumor diameter, mm; median (IQR)	34 (29–45)	36 (30–40)	0.67*
Specimen diameter, mm; Median (IQR)	35 (29–44)	38 (30–40)	0.65*
Treatment conversion, n (%)	2 (4)	16 (29)	0.0005‡
Procedure time after conversion, min; mean ± SD	N/A	44.0 (± 57.0)	N/A
Muscularis propria injury, n (%)	2 (4)	7 (13)	0.16‡
Histological pathology, n (%)			0.98‡
High-grade adenoma with villous component	4 (8)	3 (5)	
High-grade adenoma without villous component	4 (8)	6 (11)	
Low-grade adenoma with villous component	0 (0)	1 (2)	
Low-grade adenoma without villous component	5 (9)	3 (5)	
Sessile serrated lesion	4 (8)	4 (7)	
Sessile serrated lesion with dysplasia	3 (6)	2 (4)	
Adenocarcinoma (tub1)	32 (60)	34 (62)	
Adenocarcinoma (tub2)	1 (2)	1 (2)	
Other			
Histological depth, n (%)			0.26‡
Tis	31 (58)	28 (51)	
T1a	0 (0)	4 (7)	
T1b	2 (4)	3 (5)	
No malignancy	20 (38)	20 (36)	
Horizontal margin, n (%)			0.37‡
HM0	33 (62)	38 (69)	
HMX	2 (4)	2 (4)	
HM1	0 (0)	2 (4)	
No malignancy	18 (34)	13 (24)	
Vertical margin, n (%)			0.85‡
VM0	33 (62)	39 (71)	
VMX	1 (2)	1 (2)	
VM1	1 (2)	1 (2)	
No malignancy	18 (34)	14 (25)	
MLTDs used, n; mean ±SD	1.1 ± 0.4	N/A	N/A
MLTD placement success rate, n/N (%)	55/59 (93)	N/A	N/A
Lesion tissue damage at the MLTD site, n (%)	4 (8)	N/A	N/A
HM, horizontal margin; IQR, interquartile range; LST-G homo, laterally spreading tumor, granular type (homogenous subtype); LST-G mix, laterally spreading tumor, granular type (nodular mixed subtype); LST-NG FE, laterally spreading tumor, non-granular type (flat elevated subtype); LST-NG PD, laterally spreading tumor, non-granular type (pseudo-depressed subtype); MLTD, multi-loop traction device; SD, standard deviation; VM, vertical margin. *Calculated using Wilcoxon’s rank-sum test. †Calculated using Pearson’s chi-square test. ‡Calculated using Fisher’s exact test.			

### Procedure outcomes

**Fig. 3:**
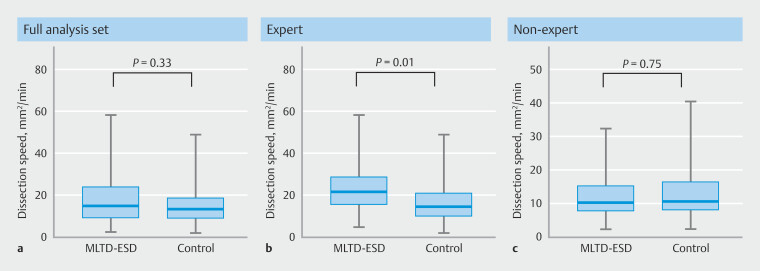
Boxplot of dissection speed. **a** Full analysis set. **b** Subgroup analysis for expert endoscopists with more than 200 clinical ESD experiences. **c** Nonexpert endoscopists with fewer than 200 clinical ESD experiences.

In the MLTD-ESD group, median dissection speed was 14.8 mm ^2^ /min (IQR 8.9–23.9), whereas in the control group, median dissection speed was 13.3 mm ^2^ /min (IQR 8.9–18.8), with no statistically significant difference (95% CI -1.89 to 5.53; P = 0.33; Fig. 3 **a** ). Procedure times were 52.0 minutes (IQR 26.5–87.0) and 55.0 minutes (IQR 40.0–80.0), respectively (95% CI -26.34 to 6.20; *P* = 0.22). The technical success rate was higher in the MLTD-ESD group, with a statistically significant difference (96.2% vs. 71.0%, 95% CI 12.3%- 38.4%, P = 0.0005). No statistically significant differences were found in en bloc resection rate (MLTD-ESD 98.1% vs. control 100%, *P* = 0.49), R0 resection rate (94.3% vs. 91.0%, *P* = 0.72), or AEs ( Table 2 ).

**Table:** **Table 2** Primary and secondary outcomes for the full analysis set.

	**MLTD-ESD (n = 53)**	**Control (n = 55)**	***P* value **
Dissection speed, mm ^2^ /min; median (IQR)	14.8 (8.9–23.9)	13.3 (8.9–18.8)	0.33*
Total procedure time, min; median (IQR)	52 (27–87)	55 (40–80)	0.22*
Technical success rate; %	96.2 %	71.0 %	0.0005†
En bloc resection rate; %	98.1 %	100 %	0.49†
R0 resection rate; %	94.3 %	91.0 %	0.72†
Adverse events; n			
Intraoperative perforation	2	3	> 0.99†
Delayed bleeding	1	1	> 0.99†
Delayed perforation	1	1	> 0.99†
IQR, interquartile range; MLTD-ESD, multi-loop traction device endoscopic submucosal dissection. *Calculated using Wilcoxon’s rank-sum test. †Calculated using Fisher’s exact test.			

PPS analysis included 51 patients in the MLTD-ESD group and 39 in the control group, after excluding treatment conversions. Dissection speed was 14.4 mm ^2^ /minute (IQR 26.0–86.0) and 14.6 mm ^2^ /minute (IQR 9.1–20.8) in the control group, with no statistically significant difference (95% CI 03.39–5.10; *P* = 0.69). Procedure times were 52.0 minutes (IQR 26.5–87.0) and 55.0 minutes (IQR 40.0–80.0), respectively, with no statistically significant difference (95% CI -19.56 to 9.21; *P* = 0.48).

### Adverse events

During this investigation, nine AEs occurred. In the MLTD-ESD group, there were two intraoperative perforations, one case of delayed bleeding, and one case of delayed perforation, all managed non-surgically. The delayed perforation may have been unrelated to ESD due to the patient’s history of total cystectomy and having been treated for an intra-abdominal abscess 1 month prior, which could have caused the free air observed on abdominal CT. In the control group, three intraoperative perforations, one case of delayed bleeding, and one case of delayed perforation were observed. One patient with early cecal carcinoma and pronounced submucosal fibrosis, whose ESD was converted to MLTD-ESD method at the 28-minute mark, developed delayed perforation on the third postoperative day. The patient underwent emergency appendectomy with ileostomy and recovered after the surgery.

### Subgroup analysis

All handovers to more experienced endoscopists occurred in primary operators who had experience with fewer than 200 ESDs; 32.1% (9/29) in the MLTD-ESD group and 61.9% (13/21) in the control group. In expert endoscopists who had experience with more than 200 ESDs, dissection speed was faster in the MLTD-ESD group, with a statistically significant difference (21.6 mm ^2^ /min, IQR 15.5–28.8 mm ^2^ /min vs. 14.4 mm ^2^ /min, IQR 9.9–21.2 mm ^2^ /min) ( *P* = 0.009). No statistically significant differences were found among nonexperts (10.3 mm ^2^ /min, IQR 7.7–15.2 vs.10.7 mm ^2^ /min, IQR 8.0–16.6 mm ^2^ /min) ( *P* = 0.75) ( Fig. 3 **b** , Fig. 3 **c** , Table 3 ), or by lesion location and size ( Table 3 ). In the control group, there was no statistically significant difference between treatment conversion cases (10.1 mm ^2^ /min, IQR 7.6–14.4 mm ^2^ /min) and non-conversion cases (14.6 mm ^2^ /min, IQR 9.1–20.8 mm ^2^ /min) ( *P* = 0.06). Among the nonexperts, dissection speed was faster in the non-handover cases compared with handover cases, with a statistically significant difference (16.3 mm ^2^ /min, IQR 11.3–18.7 vs. 8.9 mm ^2^ /min, IQR 5.9–12.0 mm ^2^ /min) ( *P* = 0.006). No statistically significant difference was found in the MLTD-ESD group (11.2 mm ^2^ /min, IQR 8.7–17.4 vs. 8.4 mm ^2^ /min, IQR 6.5–12.1 mm ^2^ /min) ( *P* = 0.09).

**Table:** **Table 3** Subgroup analysis of endoscopic submucosal dissection Bordelon speed according to tumor diameter, location, macroscopic type, and operator experience between study groups.

	**MLTD-ESD (n = 53)**	**C-ESD (n = 55)**	* *P* value
Tumor diameter, mm ^2^ /min: median (IQR)			
< 30 mm	16.5 (7.9–24.7)	9.0 (3.3–17.7)	0.11
≥ 30 mm	14.8 (8.9–24.9)	13.6 (10.0–20.0)	0.82
Tumor location, mm ^2^ /min; median (IQR)			
Cecum	12.8 (9.3–26.6)	14.4 (10.0–18.2)	0.88
Ascending colon	14.4 (10.2–21.0)	8.9 (7.6–16.7)	0.11
Transversus colon	9.4 (7.7–22.0)	12.8 (8.2–15.2)	0.19
Descending colon	12.6 (5.4–19.3)	18.2 (2.8–22.7)	0.90
Sigmoid colon	22.0 (13.2–49.5)	12.0 (10.7–20.0)	0.18
Rectum	32.5 (12.1–37.2)	26.4 (10.1–33.6)	0.60
Operator, mm ^2^ /min; median (IQR)			
Expert	21.6 (15.5–28.8)	14.4 (9.9–21.2)	0.01
Nonexpert	10.3 (7.7–15.2)	10.7 (8.0–16.6)	0.75
C-ESD, conventional endoscopic submucosal dissection; MLTD-ESD, multi-loop traction device endoscopic submucosal dissection. *Calculated using Wilcoxon’s rank-sum test.			

## Discussion

This was the first multicenter RCT to investigate efficacy of MLTD for colorectal ESD. This study involved four high-volume academic centers in Japan, including facilities unassociated with development of MLTD and 44.5% of the procedures (49/110) were performed by nonexpert as the primary operators. Both rectal and colon ESD cases were included to evaluate MLTD performance across various anatomical locations. Our study was designed to minimize subjective bias and maintain external validity.

No statistically significant difference in dissection speed was observed, even with MLTD assistance. The unexpectedly high treatment conversion rate of 29.0% observed in the control group contrasts with the 7.0% reported in CONNECT-C 18 , where conversion was limited to cases handed over to expert endoscopists after 60 minutes of difficulty in dissection. In our study, treatment conversions were allowed after 10 minutes of difficulty in dissection to prioritize patient safety, which likely prompted conversions in difficult cases and improved outcomes in the control group. In the PPS, excluding treatment conversion cases left less challenging cases in the control group, potentially masking benefits of MLTD. Variability in strategies among different operators, such as attempting submucosal dissection and delaying MLTD application even after circumferential incision, also may have influenced the outcome. We recommend attaching the MLTD immediately after circumferential incision to maximize duration of dissection plane exposure. Future studies with a rigorous conversion threshold and standardized strategies may yield different results.

There were no statistically significant differences between the two groups in en bloc and R0 resection rates, or in AEs. Results were consistent with previous Japanese traction studies, reaffirming the safety of MLTD. A limitation is lack of data about post-polypectomy electrocoagulation syndrome. Compared with the European retrospective study on double-clip traction-assisted colorectal ESD, dissection speed was slower 21 . However, our R0 resection rates exceeded 90%, with low horizontal and vertical involvement, suggesting that our institutions perform meticulous dissection.

In the subgroup analysis of experts, the MLTD-ESD group had faster dissection speeds with a statistically significant difference, an improvement not seen in less experienced nonexperts. This could be attributed to experts’ ability to more effectively utilize MLTD to optimize the surgery, considering dynamic factors such as intestinal flexures, peristalsis, tonus, and air insufflation, whereas introduction of MLTD by itself may not suffice for nonexperts to adapt to these conditions. However, these findings conflict with those from CONNECT-C, in which procedure times were shortened with traction in nonexperts, rather than experts 18 . In our study, a higher handover rate was observed among nonexperts in the control group. Our study had no handover thresholds, unlike CONNECT-C, which limited handovers to cases prolonged for 60 minutes. We observed slower dissection speed in handovers among nonexperts, indicating that this subgroup relied on expert interventions for difficult cases. This may have skewed procedure outcomes toward the control group, underestimating the benefit of MLTD for nonexperts. Although fewer handovers in the MLTD-ESD group suggest a potential benefit for nonexperts completing procedures independently, due to the lack of statistical significance of the primary endpoint, further studies are needed to confirm this finding.

Our trial has some limitations. The amount of time the MLTD was applied to the lesion was not investigated; however, in our ex vivo pilot study 6 , the average attachment time was 2.5 minutes, which should not have impacted the overall result. The open-label design of our study could not eliminate introduction of performance bias. The research was conducted exclusively in Japanese facilities, where ESD is a commonly practiced procedure, and our results may not be generalizable to Western nations where the procedure is less prevalent. In addition, availability of handovers to expert endoscopists in our study may not be generalizable to clinical practice settings where handovers are not feasible.

## Conclusions

In conclusion, this multicenter RCT of traction-assisted colorectal ESD using the MLTD device did not demonstrate a statistically significant difference in dissection speed. Our result may have been underpowered due to the high treatment conversion rate and high handover rates in the nonexpert subgroup within the control group. Further research is warranted to fully understand the impact of MLTD, particularly for less experienced practitioners.
